# Temporal analysis of vaginal proteome reveals developmental changes in lower reproductive tract of gilts across the first two weeks postnatal

**DOI:** 10.1038/s41598-019-49597-w

**Published:** 2019-09-13

**Authors:** KaLynn Harlow, Aridany Suarez-Trujillo, Victoria Hedrick, Tiago Sobreira, Uma K. Aryal, Kara Stewart, Theresa Casey

**Affiliations:** 1 Department of Animal Sciences, Purdue University, West Lafayette, Indiana USA; 2 Purdue Proteomics Facility, Bindley Science Center, Purdue University, West Lafayette, Indiana USA

**Keywords:** Differentiation, Reproductive biology

## Abstract

In swine the upper reproductive tract undergoes early postnatal development, however little is known about the lower reproductive tract. Our objective was to measure cytology and proteome of vaginal swab samples taken on postnatal day (PND) 2 and 16 in gilts to determine if temporal changes occurred in cell and protein profiles during the first two weeks after birth. The posterior vagina was swabbed using a cytology brush on PND 0, 2 and 16 and slides were prepared. The proportion of anuclear and superficial cells increased and parabasal decreased (P < 0.05) from PND 0 to 16. Proteins isolated from vaginal swabs taken on PND 2 and 16 from six gilts across three litters were measured using LC-MS/MS. Over 1500 proteins were identified, with 881 differentially expressed (P-adj < 0.05) between PND 2 and 16. One-third of proteins upregulated between days were categorized as secreted, including lipocalins. Categories enriched by downregulated proteins included *cell-cell adherens junction, translation* and *ER to Golgi vesicle-mediated transport*, and reflected increased cornification of stratified epithelium and thus mirrored changes in cytology. Changes in cytology and proteome over the first two weeks after birth support that the porcine vagina continues to develop postnatal.

## Introduction

A significant amount of female reproductive tract development occurs in the early postnatal period in swine^1–7^. There is interest in studying effects of early nutritional or xenobiotic exposures on reproductive tract development and the relationship to fertility and health in swine. However animals must be euthanized to analyze histology and isolate RNA or proteins from the uterus, and so the impact of perinatal exposure cannot be followed in the same animal across time. Thus, there is a need for a model that enables researchers to measure the effect of an early perinatal exposure on reproductive tract developmental changes across time and in relation to long-term reproductive outcomes.

We hypothesized that, similar to the uterus, the vagina continues to develop in the first two weeks postnatal in gilts and nutritional environment influences developmental changes. Recently we reported that lipids captured by swabbing vagina of neonatal gilts with cytology-pap brush can be used for lipidome analysis and that perinatal diet of gilts significantly affected lipid composition of vagina^8^. We envisioned that developmental changes in the female reproductive tract could also be measured non-invasively by obtaining vaginal cell samples with a pap brush for proteome analysis across time periods. The objectives of this study were to: (1) Determine if there were differences in vaginal cytology between postnatal day (PND) 0, PND 2 and PND 16 in suckling gilts; and (2) Measure proteins from vaginal swabs taken on PND 2 and PND 16 using label free shotgun proteomics to determine if proteome changes over the first two weeks postnatal.

## Results

### Distribution of cells obtained with vaginal swab on PND 0, PND 2 and PND 16

**Figure 1:**
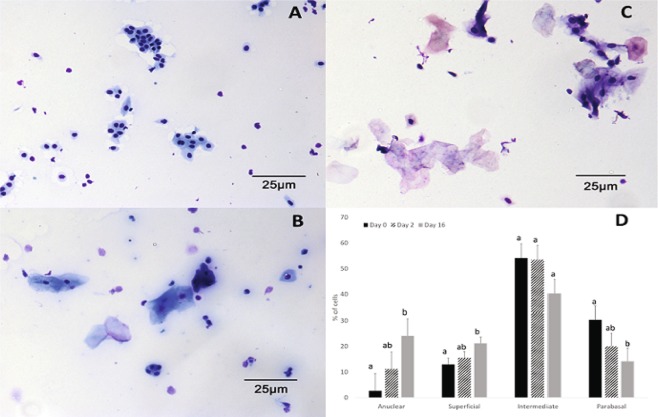
Cytology smears prepared from vaginal swabs taken from gilts on (**A**) PND 0, (**B**) PND 2, and (**C**) PND 16. (**D**) Distribution of stratified epithelium subtypes in cytology smears prepared from gilts in PND 0, PND 2 and PND 16. Different letters indicate difference at P < 0.05.

Cytological smears were prepared from vaginal swabs taken from gilts on PND 0, PND 2 and PND 16. Smears contained primarily epithelial cells, either isolated or in aggregates (Fig. 1A–C). Quantification of stratified cell-subtypes found a greater (P < 0.05) proportion of anuclear and superficial cells and a lower (P < 0.05) proportion of parabasal cells in smears prepared from PND 16 versus PND 0 samples. There was no significant difference in proportion of cell-subtypes between PND 2 and PND 0 or PND 2 and PND 16, however numerical differences between PND 2 and PND 16, showed similar trends as between PND 0 and PND 16 (Fig. 1D). Examination of histological sections of vagina cross sections showed areas across the posterior region where multiple layers of the stratified epithelium were removed by pap brush during swabbing. In areas the epithelium appeared intact, histological differences between the days supported changes in cellular subtypes measured in cytology smears. In particular, there were more cell layers in the stratified epithelium on PND 16 (6–7 layers) than on PND 0 or PND 2 (3–4 layers), and the apical layer was composed of anuclear flattened cells on PND 16. Whereas nucleated squamous to cuboidal shaped cells were most apical on PND 2 and PND 0. Quantification of number of layers of stratified epithelium on histology slides was not possible, as there was a lack of certainty in calling areas as swabbed versus intact.

### Temporal effects on neonate vaginal proteome in the first two weeks postnatal

**Figure 2:**
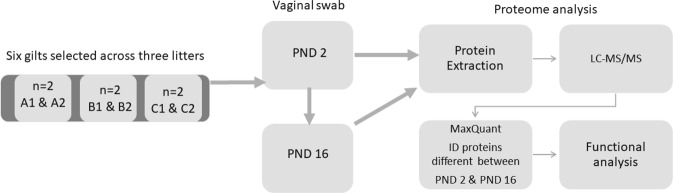
Schematic of study design and workflow for temporal analysis of vaginal proteome between PND 2 and PND 16.

Six median sized gilts were selected across three litters (n = 2 gilts/litter) for temporal proteome analysis (Fig. 2). Label-free shotgun proteomics using liquid chromatography-tandem mass spectrometry (LC-MS/MS) analysis detected 1823 proteins at least once across all samples. Of these, 1554 proteins were detected with high confidence, as defined by expression in at least four animals across three litters, on at least one day. Using Uniprot and GenPept *Sus scrofa* IDs for functional annotation analysis resulted in loss of approximately half of the dataset, with only 806 proteins mapping to known DAVID IDs (DAVID Bioinformatics Resources 6.8). In order to maintain robustness of data set, gene name assigned to protein ID was submitted to Biomart and converted to 1361 human specific Ensembl gene IDs (http://useast.ensembl.org/biomart)^9^ (Supplemental Table S1). The remaining 194 proteins did not map to human Ensembl gene IDs because they were uncharacterized proteins or unique to *Sus scrofa* (for example LGB; Supplemental Table S2). The 1361 human Ensembl IDS were used for all subsequent DAVID analyses. Annotations of proteins unique to *Sus scrofa* were done individually using GeneCards.

The top one-third of proteins expressed (mean expression across both time points) were submitted to DAVID for functional analysis (Table 1 and Supplemental Table S3), and revealed 17 proteins categorized as *intermediate filaments*, with nine being keratins. Seven proteins were categorized as *cornified envelope* including CNFN (cornifelin), EVPL (envoplakin), IVL (Involucrin) and SPRP (small proline rich protein). In addition, 57 proteins were categorized as structural constituents of ribosomes and 29 as actin cytoskeleton proteins (Table 1). *Cell redox homeostasis* was enriched with ten of the most highly expressed proteins and included four peroxiredoxins (PRDX), catalase (CAT), superoxide dismutase (SOD1), and thioredoxin (TXN). Twenty-four proteins enriched the category *innate immune response* including BPIFA, Peptidoglycan Recognition Protein 1 (PGLYRP1), Lipocalin 2 (LCN2) and four s100A proteins (S100A7, S100A8, s100A9, S100A12).

**Table 1:** Representative categories enriched with top third of most abundant proteins (mean expression across PND 2 and PND 16) isolated from vaginal swabs taken from neonatal gilts.

Term	Count	%
GO:0005882~intermediate filament	17	3.6
GO:0006096~glycolytic process	10	2.1
GO:0000302~response to reactive oxygen species	10	2.1
GO:0045087~innate immune response	24	5.1
GO:0001533~cornified envelope	7	1.5
GO:0003735~structural constituent of ribosome	57	12.3
GO:0015629~actin cytoskeleton protein	29	6.2

**Figure 3:**
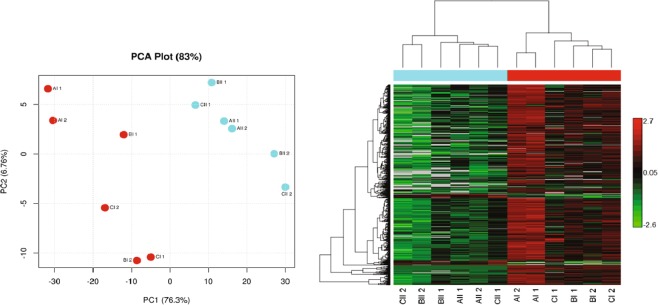
(A) Principle component analysis (PCA) and (B) hierarchical cluster analysis with heat map for protein analysis separated by time point. Red clusters on PCA plot and in hierarchical cluster analysis indicate first time point (I) on PND 2. Light blue indicates second time point (II) on PND 16. For the heat map, animal IDs with associated time point are on the bottom. Within the heat map, green indicates downregulation and red indicates upregulation.

**Figure 4:**
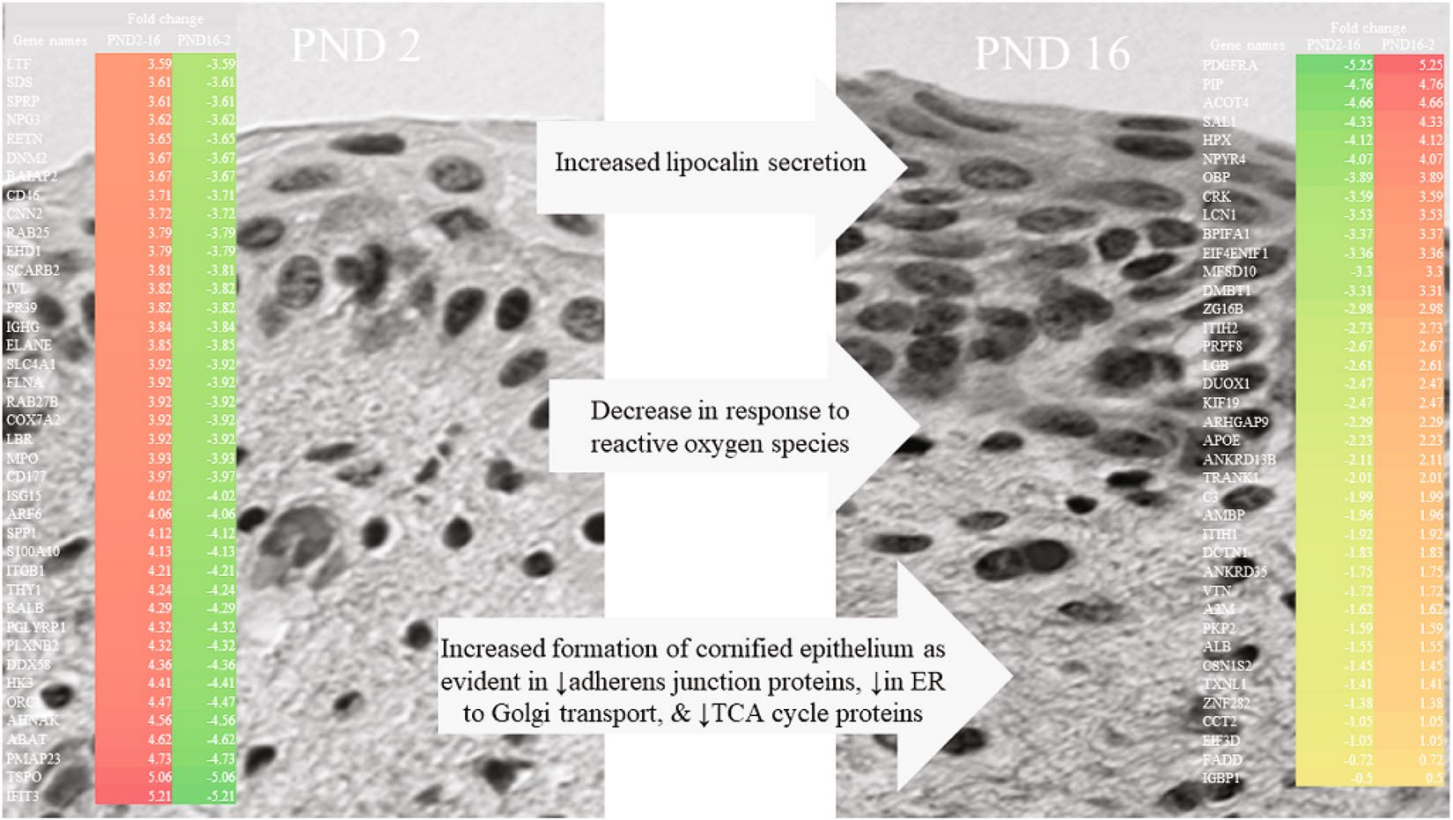
Schematic illustrating changes in gilt vaginal swab proteome between PND 2 and PND 16 highlighting enriched categories and the 39 of the 44 proteins upregulated and top 40 downregulated between PND 2 and PND 16.

Principle component and hierarchical cluster analysis revealed distinct clusters of samples by postnatal day (Fig. 3). There were 25 proteins expressed only on PND 2, and included NADH dehydrogenase flavoprotein 1 and histone cluster 1 H1, and one protein unique to PND 16 (Table 2), with heat map demonstrating that the vast majority of differentially expressed proteins (P < 0.05; 818; Table 3) were down regulated (773) between day 2 and 16 postnatal (Fig. 3). About one-third of the 44 upregulated proteins (*P* < 0.05; Fig. 3) were categorized as secreted or extracellular proteins including prolactin induced protein (PIP), salivary lipocalin 1 (SAL1), oderant binding protein (OBP), and beta-lactoglobulin (LGB). PIP also has antimicrobial properties along with three other upregulated proteins, BPIFA, C3, and DMBT1. Six proteins up regulated between day 2 and 16 were categorized as negative regulators of endopeptidase, and included alpha 2 microglobulin, AMBP, ITIH1 and ITIH2, vitronectin and alpha 2 microglobulin (Fig. 4).

**Table 2:** Proteins Unique to PND 2 or PND 16.

Gene symbol	Protein name
**Detected only in PND 2 swab samples**	
LAMTOR1	Late endosomal/lysosomal adaptor; MAPK and MTOR activator 1
SPTLC2	Serine palmitoyltransferase long chain base subunit 2
OXSR1	Oxidative stress-responsive 1 protein
SSR4	Signal sequence receptor subunit 4
MYZAP	Myocardial zonula adherens protein
IDH3G	Isocitrate dehydrogenase [NAD] subunit gamma
SCAMP1	Secretory carrier-associated membrane protein 1
STX12	Syntaxin-12
SEC. 24 C	SEC. 24 homolog C; COPII coat complex component
LAMP-1	Lysosome-associated membrane glycoprotein 1
FIS1	Mitochondrial fission 1 protein
SLPI	Secretory leukocyte protease inhibitor
CSRP1	Cysteine and glycine rich protein 1
NDUFV1	NADH dehydrogenase [ubiquinone] flavoprotein 1
HIP1R	Huntingtin interacting protein 1 related
KYAT3	Kynurenine aminotransferase 3
RAB5A	Ras-related protein Rab-5A
FGFBP1	Fibroblast growth factor binding protein 1
COPG1	Coatomer subunit gamma
SMC3	Structural maintenance of chromosomes protein
ALOX5AP	Arachidonate 5-lipoxygenase-activating protein
HIST1H1E	Histone cluster 1 H1 family member e
PDS5A	PDS5 cohesin associated factor A
ITGB2	Integrin beta
**Detected only in PND16 swab samples**	
CIRH1A	Cirhin (Fragment)

**Table 3:** Representative categories enriched with proteins downregulated between PND 2 and PND 16.

Term	Count	%
GO:0005913~cell-cell adherens junction	102	15.4
GO:0006099~tricarboxylic acid cycle	14	2.1
GO:0006412~translation	31	4.7
GO:0006888~ER to Golgi vesicle-mediated transport	25	3.7
GO:0006457~protein folding	24	3.6
GO:0000302~response to reactive oxygen species	10	1.5
GO:0045087~innate immune response	26	3.9

Of the 773 proteins down regulated between day 2 and 16, 692 were assigned human Ensembl gene IDs in Biomart. Functional annotation analysis in DAVID categorized 15% (102 proteins) as *cell-cell adherens junction* proteins, 2% (14 proteins) in biological processes associated with the *tricarboxylic acid cycle*, and nearly 5% played functional roles in *translation* (Supplemental Table S4; Fig. 4). Twenty-five proteins were categorized in the biological process *ER to Golgi vesicle-mediated transport*, twenty-four in *protein folding*, and ten in *response to reactive oxygen species* to include six peroxiredoxins and thioredoxin.

## Discussion

The mature vagina is composed of a non-keratinized stratified squamous epithelium, which protects the underlying tissues from mechanical, chemical, and microbial insults. The multilayered stratified squamous epithelium contains several distinct layers: the mitotically active basal layer, the suprabasal layer, and a superficial layer of flattened cornified cells^10^. Cytology smears prepared from vaginal swabs showed that the distribution of these cellular subtypes changed between days 0 and 16 postnatal, and changes support the continued development of the posterior vagina during the first two weeks postnatal. Postmortem histological analysis of gilts swabs taken from the same gilts found that there were more cellular layers in the stratified epithelium of the PND 16 versus than PND 2 and PND 0 gilts. Anuclear cells were the primary cells present in the most apical layer of intact stratified epithelial layers on PND 16, but absent on PND 0.

The proteome signature of the most highly expressed proteins across both time points supported that proteins captured on swabs were components of epithelial cells lining the lumen of the vagina. The enrichment of seven proteins in *cornified envelope* reflected vaginal histomorphology of stratified squamous epithelium, with corneodesmesomes located in the transitional region immediately below the most apical cells^10^. Keratins were also among the most highly expressed proteins across both time points and included KRT4, KRT5 and KRT13, which are expressed by vaginal basal and suprabasal epithelial cells^10^. Moreover, the enrichment of proteins in glycolysis and gluconeogenesis related categories may reflect the relatively unique intracellular glycogen stores of vaginal epithelial cells^10^.

The squamous epithelium of the posterior vaginal serves as a barrier to pathogen invasion and in mechanical protection during copulation and birth. In the mature vagina the most apical cells are comprised of dead flattened cells that have undergone a terminal cell differentiation program called cornification^10^. During the process of cornification, nuclei and intracellular organelles are extruded and functional DNA and RNA is depleted, and thus there is no new synthesis of proteins. Terminally differentiated cornified cells do not maintain robust intercellular junctions, and although the capacity of cells to actively respond to microbial exposure is diminished, the vaginal stratum corneum mounts effective defense against invasive microbial infections. The loosely connected glycogen-filled cells of the vaginal stratum corneum are permeable to microorganisms and molecular and cellular mediators of immune defense, and foster an environment that encourages growth of *Lactobacilli* and retains mediators of acquired and innate immunity to maintain vaginal health^10,11^. In our analysis, the enrichment of proteins in *innate immune response* ontology reflected this environment, with multiple proteins detected in vaginal swabs on days 2 and 16, including calprotectin (S100A8/S100A9), also found in proteomic analysis of secretions from the upper and lower genital tract of women^12^. *Lactobacilli*, the primary commensal bacteria found in the vagina of humans and swine, function as a barrier to pathogens in part by production of lactic acid and hydrogen peroxide^11,13,14^. Proteins highly expressed on both PND 2 and PND 16 regulated responses to reactive oxygen, with four proteins (PRDX, CAT, SOD1, TXN) functioning to regulate detoxification pathways related to hydrogen peroxide. Thus, enrichment of this category likely reflected *Lactobacilli* colonization of the neonatal porcine vagina.

Basal and suprabasal layers of stratified vaginal epithelium lie below the cornified layers of the vagina, and are held together by proteins that form desmosomes, tight junctions, and adherens junctions^10,11^. The protein signatures related to cell-cell adhesion reflected that basal layers were captured with vaginal swabs. Moreover, the decrease in adherens junction protein abundance from day 2 to 16 postnatal likely reflected that proportionally more terminally differentiated (cornified cells) versus basal and suprabasal cells were sampled on day 16. Proteins involved in the tricarboxylic acid cycle, translation, and protein folding were also down regulated between postnatal days 2 to 16. Proteins present on PND 2 but absent on PND 16 were linked to energy metabolism and nucleosome structure, including NADH Dehydrogenase Flavoprotein 1 and Histone Cluster 1 H1. NADH Dehydrogenase Flavoprotein 1 is a well-known component of the mitochondrial respiratory chain, and histones are responsible for the compaction of genomic DNA. These changes in abundance of proteins likely reflect extrusion of DNA containing nuclei and intracellular organelles, and thus the depletion of functional mitochondria and DNA and RNA from vaginal cells as they undergo cornification to terminally differentiate^10^. Thus, these proteome signatures further support the more differentiated state of the vagina of two-week old versus two-day old gilts.

Proteins that were more abundant on PND 16 also likely reflected a greater degree of vaginal differentiation and included antimicrobial proteins, protease inhibitors, and lipocalins. Lipocalin proteins are small extracellular proteins that transport hydrophobic compounds (steroids, bilins, retinoids, and lipids) in aqueous biological fluids^15^; those increased between PND 2 and 16 included SAL1, OBP, LGB, LCN1, and AMBP, which is cleaved into the lipocalin Alpha-1-microglobulin. Odorant binding proteins (OBP) are small, soluble proteins that are present in the olfactory apparatus as well as in biological fluids such as saliva, urine or vaginal discharge, and are able to bind pheromones^15^. OBP are assumed to be directly involved in chemical communication and in the pre-mating recognition process. Salivary lipocalin (SAL1) is the most abundant OBP isolated from submaxillary glands of mature male pigs. SAL1 binds to pheromonal steroids and appears to play a key role in the standing reflex in the sow and the boar’s libido. SAL1 protein has also been detected in the porcine uterine luminal fluids, and is proposed to play a role in establishment of pregnancy in pigs^15^.

## Conclusion

Temporal changes in proteins harvested from gilt vaginal swabs support that similar to the uterus the lower reproductive tract continues to develop the first two weeks postnatal in swine. Changes in proteome signature were indicative of cornification of stratified epithelium and mirrored changes in cytological distribution of cells captured on brushes. The greater abundance of lipocalin proteins in two-week old gilts also reflected a more differentiated state. Proteins isolated from vaginal swabs were similar to those found in women^12^, which corroborated previous studies highlighting the similarities between mature human and porcine vaginal protein expression. Together with similar histomorphology between porcine and human vagina^16^, our findings support the potential that swine may serve as a good model for studying effects of perinatal exposures on reproductive tract development, with vaginal swab proteome serving as a noninvasive means of tracking changes across time.

## Materials and Methods

### Animals and study design

Prior to start of study, the Purdue Animal Care and Use Committee (Protocol #1605001416) reviewed and approved all procedures involving animals. Standard farrowing protocols for the Purdue University Animal Sciences Research and Education Swine facility were followed. For cytology analysis, vaginal swabs were taken on PND 0, 2 and 16 from three gilts on each day. Using a cytology brush (Puritan 2196 Removable Stiff Bristle Tip Brush; Quick Medical; Issaquah, WA) swabs were taken by inserting the tip of the brush into the vulva angled dorsally at 45°. Once inserted to the base of the bristles, the brush was rotated 360° against the vaginal surface. Two consecutive swabs were collected from each animal. To examine histomorphology following swabs, gilts were euthanized using CO_2_ in accordance with the AVMA Guidelines for the Euthanasia of Animals_,_ and dissected in order to remove the vagina.

On PND 2 and PND 16 vaginal swabs were taken from gilt six gilts across three litters (Fig. 2), and brush was placed in a 15 ml sterile polypropylene conical tube (Falcon™, Fisher Scientific, San Jose, California) with 2 ml of 50 mM Hepes/ KOH, 1 mM PMSF, 5% glycerol, 1 mM EDTA and 5 mM beta-mercaptoethanol and transported on ice to the Purdue Proteomics Facility for proteomic analysis.

### Cytology and histology analysis

Following vaginal swab, cytology brush was smeared across a glass slide. Material on slide was air dried, and then stained with manual staining solutions (Protocol Hema 3 Solutions, Fisher Scientific, Hampton, NH). Cytology smears (n = 2/piglet/time point) were evaluated using a Media Cybernetics Evolution MP Color camera under light microscopy at 400X. Approximately 200 cells/slide were categorized as parabasal (~10 µm cells with big nuclei), intermediate (15–20 µm cells), superficial (>20 µm cells with small nuclei) or anuclear (>20 µm cell without nuclei).

The length of vagina between the uterine cervix and the vulva was dissected, fixed in 10% buffered formalin for 24 h and paraffin embedded. The total length of each vagina was divided in three even portions and labelled as posterior (tissue closer to the vulva), medial (middle portion) and anterior (tissue closer to the cervix). Five micron sections of posterior portion were placed on slides and processed for staining with hematoxylin and eosin (H&E). Histology sections were evaluated using a Media Cybernetics Evolution MP Color camera under light microscopy at 200X, respectively.

### Statistical analysis of cellular distribution

PROC MIXED procedure from SAS software (version 9.4, SAS Institute Inc., Cary, NC) was used, followed by Tukey post hoc test, to compare the percentage of each cell type by day. Values were considered significant when *P* < 0.05.

### Proteomic analysis (schematic of work flow figure 2)

#### Protein extraction and LC-MS sample preparation

The cytology brush was submerged in 2 mL of 20 mM PBS buffer, pH 7.5, 1 mM EDTA, 5% glycerol, 0.5 mM DTT (dithiothreitol) and 2 mM freshly prepared phenylmethylsulfonyl fluoride (PMSF), and incubated at room temperature for 1 h with continuous shaking. The extracted proteins were precipitated using 5 volume of cold (−20 °C) acetone, and incubated overnight at −20 °C. Samples were centrifugation at 14,000 rpm for 15 min at 4 °C to pellet precipitated proteins, and pellets were washed in 80% acetone (−20 °C). To solubilize proteins, 40 µL of 8 M urea was added to pellets and samples were vortexed continuously for 1 h at room temperature. To remove undissolved pellets, samples were centrifuged at 14,000 rpm for 15 min at 4 °C, and concentration of protein in supernatant was measured with bicinchoninic acid (BCA) assay using BSA standards. Incubation with 10 mM dithiothreitol (DTT) at 50 °C for 45 min was used to reduce protein (50 µg). Samples were cysteine alkylated with 20 mM iodoacetamide (IAA) by incubating for 45 min at room temperature in the dark. To remove residual IAA, samples were incubated with 5 mM DTT for 20 min at 37 °C. Sequencing grade trypsin and Lys-C mix from Promega was added at a 1:25 (w/w) enzyme-to-protein ratio at 37 °C overnight. Following manufacturer’s protocol the digested peptides were cleaned using C18 silica micro spin columns (The Nest Group Inc.), vacuum dried and re-suspended in 3% acetonitrile and 0.1% formic acid. The BCA assay was used to determine peptide concentration, and concentration was adjusted to 0.2 µg/µL.

#### LC-MS/MS data acquisition

Reverse-phase LC-ESI-MS/MS was used to analyze samples on the Dionex UltiMate 3000 RSLC nano System coupled to the Q Exactive High Field (HF) Hybrid Quadrupole Orbitrap MS and a Nano- electrospray Flex ion source (Thermo Fisher Scientific). Peptides were loaded onto a trap column (300 μm ID × 5 mm) packed with 5 μm 100 Å PepMap C18 medium, and then separated on a reverse phase column (50-cm long × 75 µm ID) packed with 3 µm 100 Å PepMap C18 silica (Thermo Fisher Scientific). The positive ion mode was used with 120 min LC gradient. Formic acid (0.1% FA) in water was used for solvent A (mobile phase) and 0.1% FA in 80% acetonitrile was solvent B. Peptides were loaded to the trap column (50 °C) in 100% of solvent A for 5-min at 5 µl/min flow rate, and eluted with a linear 80-min gradient of 5–30% of solvent B. It was changed to 45% of solvent B at 91 min, 100% of solvent B at 93-min, when the gradient was held for 7 min, and then reverted back to 95% of solvent A at 100-min. The column was equilibrated at 95% of solvent A for 20 min. A flow rate of 300 nl/min was used to separate peptides from the analytical column. Columns were washed 2 × with 30-min linear gradient of 5–45% of B after each 120-min sample run. Standard data-dependent mode was used for the mass spectrometer, and data were acquired using the Top20 data-dependent MS/MS scan method. Collection of data for full scan MS spectra was done using a range of 400–1,600 m/z, and a maximum injection time of 100 milliseconds. The resolution was set at 120,000 at 200 m/z and a spray voltage of 2 and AGC target of 3 × 10^6^ was used. High-energy C-trap dissociation (HCD) with the normalized collision energy of 27 eV was used for fragmentation of precursor ions. To acquire MS/MS scans, a resolution of 15,000 at m/z 200 with an ion-target value of 1 × 10^5^ and a maximum injection of 20 milliseconds was used. The dynamic exclusion was set at 15 s. Instrument performance was insured by calibrating with mix solution (Thermo Scientific) prior to batch runs, and then every 72 h, and evaluated using *E. coli* digest (Sigma).

#### Data analysis

MaxQuant software (v. 1.6.0.16)^17–19^ with its built-in Andromeda search engine was used for analysis of raw LC-MS/MS data. The MS/MS spectra were searched against the *Sus Scrofa* protein database from Uniprot (downloaded on August 21, 2018), for protein identification. Parameters for database search were set in the following way: minimum length, 7 amino acids, precursor mass tolerance was 10 ppm and MS/MS fragment ions tolerance was 20 ppm. Moreover, database search was performed with trypsin and LysC enzyme specificity, with ≤2 missed cleavages; variable and fixed modifications were defined as oxidation of methionine and N-terminal acetylation, and carbamidomethylation of cysteine, respectively. For peptide quantitation ‘unique plus razor peptides’ were used, and the false discovery rate (FDR) was set at 0.01 for both peptide and protein identification. Label-free quantification (LFQ) intensities were used to calculate relative protein abundance, and data searched with “match between runs” option. When peptides were shared between two proteins, these were combined and reported as one protein group. Proteins matching to the reverse database were removed.

Data visualization, log transformation and correlations coefficients of samples were calculated using LFQ intensities in InfernoRDN^20^. Data normality and homogeneity of variances were confirmed prior to running analysis of variance (ANOVA) and Tukey adjustment in R (https://www.r-project.org/) to identify differentially abundant proteins between vaginal swabs taken on postnatal day 2 versus day 6 analysis using repeated measured. Differences between days with adjusted-P ≤ 0.05 were considered significant. Functional annotation analysis of proteins was done using NIH Database for Annotation, Visualization and Integrated Discovery (DAVID) v6.8^21,22^. GeneCards^23^ and Uniprot^24^ databases were used to define protein function. Data were deposited in the public repository MassIVE and can be found with the following ID MSV000083361.

## Supplementary information

Table S1, Table S2, Table S3, Table S4
